# The Self-Organization of Marine Microbial Networks under Evolutionary and Ecological Processes: Observations and Modeling

**DOI:** 10.3390/biology11040592

**Published:** 2022-04-13

**Authors:** Zhenghua Liu, Jianjun Wang, Delong Meng, Liangzhi Li, Xueduan Liu, Yabing Gu, Qingyun Yan, Chengying Jiang, Huaqun Yin

**Affiliations:** 1 School of Minerals Processing and Bioengineering, Key Laboratory of Biometallurgy of Ministry of Education, Central South University, Changsha 410083, China; liuzhenghua2017csu@163.com (Z.L.); delong.meng@gmail.com (D.M.); 205601006@csu.edu.cn (L.L.); xueduanliu@yahoo.com (X.L.); guyabing0207@163.com (Y.G.); 2 State Key Laboratory of Lake Science and Environment, Nanjing Institute of Geography and Limnology, Chinese Academy of Sciences, Nanjing 210008, China; jjwang@niglas.ac.cn; 3 School of Environmental Science and Engineering, Sun Yat-sen University, Guangzhou 510006, China; yanqy6@mail.sysu.edu.cn; 4 Institute of Microbiology, Chinese Academy of Sciences, Beijing 100101, China; jiangcy@im.ac.cn

**Keywords:** microbial ecology, microbial community, network, self-organization, marine

## Abstract

**Simple Summary:**

The properties and structure of ecological networks in marine microbial communities determine ecosystem functions and stability; however, the principles of microbial network assemblages are poorly understood. In this study, we revealed the influences of species phylogeny and niches on the self-organization of marine microbial co-occurrence networks and provided a mathematical framework to simulate microbial network assemblages. Our results provide deep insights into network stability from the perspective of network assembly principles and not just network properties, such as complexity and modularity.

**Abstract:**

Evolutionary and ecological processes are primary drivers of ecological network constrictions. However, the ways that these processes underpin self-organization and modularity in networks are poorly understood. Here, we performed network analyses to explore the evolutionary and ecological effects on global marine microbial co-occurrence networks across multiple network levels, including those of nodes, motifs, modules and whole networks. We found that both direct and indirect species interactions were evolutionarily and ecologically constrained across at least four network levels. Compared to ecological processes, evolutionary processes generally showed stronger long-lasting effects on indirect interactions and dominated the network assembly of particle-associated communities in spatially homogeneous environments. Regarding the large network path distance, the contributions of either processes to species interactions generally decrease and almost disappear when network path distance is larger than six. Accordingly, we developed a novel mathematical model based on scale-free networks by considering the joint effects of evolutionary and ecological processes. We simulated the self-organization of microbial co-occurrence networks and found that long-lasting effects increased network stability via decreasing link gain or loss. Overall, these results revealed that evolutionary and ecological processes played key roles in the self-organization and modularization of microbial co-occurrence networks.

## 1. Introduction

The alterations of species interactions in ecological network, whether direct and indirect, are giving rise to cascading extinctions [1,2]. Thus, there is an urgent need to explore the determinants of species interactions and the ways that they drive assemblage dynamics of ecological networks. Direct species interactions are widely considered to be constrained by evolutionary and ecological processes [3,4,5,6,7,8]. For examples, phylogeny and optimal niches or functional traits, the proxies of evolutionary and ecological processes, respectively, are key predictors of species interactions [9,10,11,12,13]. However, the question of which process plays the dominant role in constructing species ties is still understudied. Additionally, few studies pay attention to the relative contributions of these processes to indirect species interactions within ecological networks.

Ecological networks are inherently hierarchical [14], and thus the assembly driven by evolutionary and ecological processes can be characterized from various network levels, such as nodes, sub-networks (e.g., motif [15] and module), and the whole network. The metrics at node level, e.g., the degree [16] and within- and among-module connectivity [17], could measure the topological role of species in ecological networks [18,19,20]. However, less is known on how such topological properties are jointly driven by the phylogeny and niches of these species. At the sub-network level, motifs are common network backbones, and their elements may have similar traits or shared evolutionary histories [15,21,22]. Modules are purely identified by mathematical methods, such as the algorithms of greedy modularity optimization [23] and short random walks [24], but constrained by evolutionary and ecological processes revealed by previous reports on pollination networks, seed-dispersal networks and microbial networks [17,20,25,26]. At the whole network level, both processes are observed as primary drivers that shape microbial networks [27,28,29], but how these processes shape network global properties, such as self-organization and modularity, is less reported.

Such self-organization and modularization of ecological networks could be essentially revealed by establishing a mathematical model and simulating network assembly. In order to construct ecological networks, there are the three key features that must be re-emphasized from previous studies [30]: (i) the aggregations of nodes are dynamic; the network can gain or lose them, and they can connect or disconnect with each other [31], meaning that species have dynamic connections with other species. (ii) The nodes harbor specific properties, such as phylogeny and niches. (iii) External forces such as environmental disturbance can also act on network structures. Moreover, we expect that evolutionary and ecological processes should further take into account the self-organization of ecological networks. It is critical for us to understand how ecological networks respond to environmental changes and access network stability beyond posteriori knowledge.

Here, our objective is to study the evolutionary and ecological processes that shape marine microbial co-occurrence networks across multiple network levels. Based on global ocean metagenomic data from the TARA project across three depth layers and two size fractions [32], we analyzed the effects of phylogeny and niches on microbial co-occurrence networks across the levels of nodes, motifs, modules and the whole network, and further developed a novel mathematical model for simulating the self-organization of microbial networks based on a scale-free network [33,34]. We focused on three key questions: (1) How are direct and indirect interactions in microbial co-occurrence network constrained by both evolutionary and ecological processes? (2) What is the relative importance of evolutionary and ecological processes in determining co-occurrence network assemblages? (3) How do the characteristics of these two processes underpin co-occurrence network stability under environmental disturbance?

## 2. Materials and Methods

### 2.1. Datasets

We obtained meta data from the global ocean data of TARA project [32], and the samples were grouped into six subsets according to the three depth layers (SRF: surface water layer; DCM: deep chlorophyll maximum layer; MES, mesopelagic zone) and two size fractions (free-living: 0.22–3.0 μm; particle-associated: 0.80–5.0 μm) [32,35]. For the consistency of downstream analyses, the read depths of each sample of metagenomic 16S rRNA tags were rarefied to 10,000. Phylogenetic tree of representative OTUs was extracted from SILVA 128 database (QIIME release) [36] based on 97% similarity.

### 2.2. Network Construction, Motif and Latent Space Analyses

In each sub-dataset, OTUs observed in over a half of the samples were selected for network constructions. The correlations between OTUs were inferenced via SparCC tool [37,38,39] and carried out by ‘sparcc’ function (iter = 100, inner_iter = 100, th = 0.3) [38]. The correlations between OTUs that were no less than 0.65 were kept for network constructions. The memberships of nodes were identified by the fast-greedy modularity optimization method [23] via ‘cluster_fast_greedy’ function. The frequencies of each node for motif positions in undirected networks were counted using Simmons’s method, which was conducted via the ‘mcount’ function (six_node = TRUE) [40]. For each node in the network with the largest connectivity, the positions within the latent space in two dimensionalities and the probabilities of clustering memberships were calculated by the Variational Bayes Latent Position Cluster Model (VBLPC) [41]. To determine the optimal number of groups, we searched the minimum Bayes information criterion (BIC) with desired group scopes (Figure S1A). The latent space distances between OTUs were calculated as Euclidean distances. These network analyses were conducted in R version 3.6.1 with the packages of ‘SpiecEasi’ V1.0.7, ‘iGraph’ V1.2.4.2, ‘bmotif’ V1.0.0 and ‘VBLPCM’ V2.4.5 [38,40,41,42].

### 2.3. Statistical Analyses

Phylogenetic tree was transferred into pairwise distance matrix via ‘cophenetic’ function. The optimal habitat value, as a niche for each out, was calculated based on environmental variables [43]. Between-OTU niche differences were calculated as Euclidean distances, and between-OTU differences in topology properties of network motifs and module levels were calculated as Bray distance based on motif position frequencies and the probabilities of clustering memberships. We used multiple logistic regression in the distance matrices with 999 permutations [44] to quantify the effects of phylogeny and niches on network adjacent matrices (binary data). Similarly, we also used multiple linear regression in distance matrices to examine the regression coefficients of pairwise phylogeny and habitat distance against pairwise motif or module differences. These analyses were performed with packages of ‘base’, ‘vegan’ V2.5–6 [45] and ‘ecodist’ V 2.0.1 [46].

**Figure 1:**
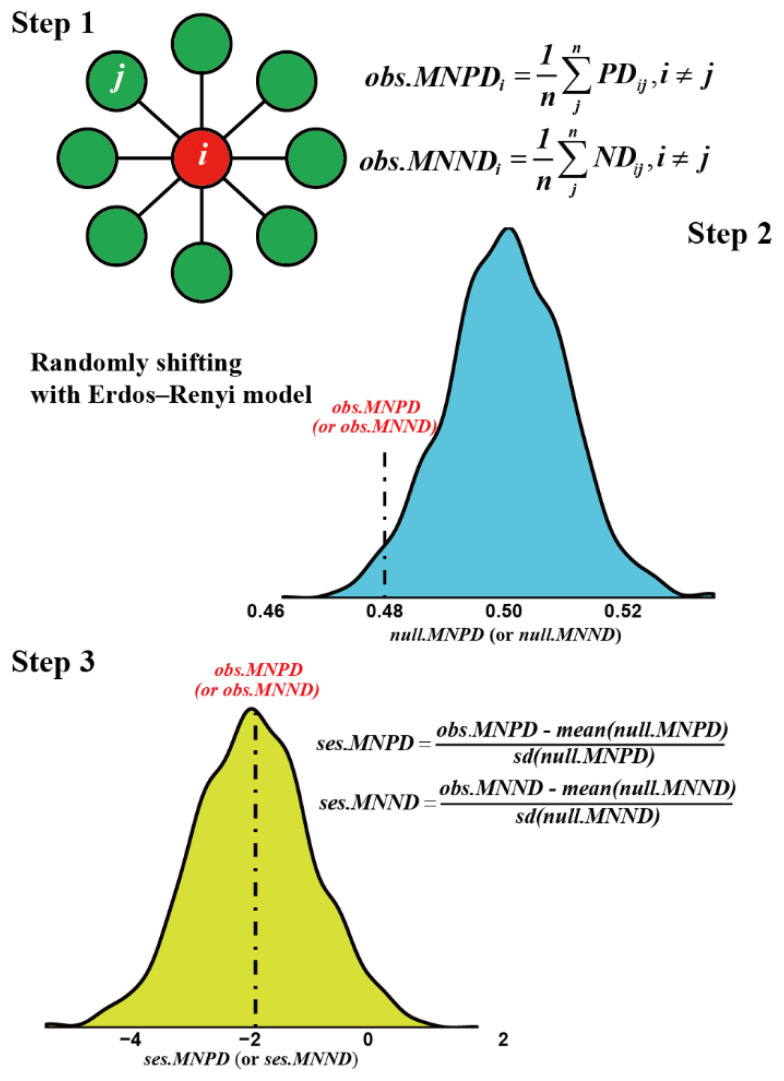
The flowchart of the summarizing procedure for estimating mean neighbor phylogeny and niche distance (*MNPD* and *MNND*) at node level. First, the observed *MNPD* and *MNND* (*obs.MNPD* and *obs.MNND*) were quantified based on empirical networks. Second, the links of empirical networks were randomly shifting with the Erdos–Renyi model, and null distributions of *MNPD* and *MNND* (*null.MNPD* and *null.MNND*) were generated. Third, the standardized effect sizes of *MNPD* and *MNND* (*ses.MNPD* and *ses.MNND*) were calculated based on observed and null values. The absolute values of *ses.MNPD* (*ses.MNND*) larger than two show statistical significance (two sides, *p* < 0.05).

To estimate species preference in phylogeny and niches within networks, we proposed the novelty network-based metrics (Figure 1), that is, the mean neighbor phylogeny distance (MNPD) and mean neighbor niche distance (MNND), respectively. These two metrics were calculated by the following formula:

(1) $$MNPD_{i}=\frac{1}{n}\times \overset{n}{\underset{j}{\sum }}PD_{ij}\;i\neq j$$

and:

(2) $$MNND_{i}=\frac{1}{n}\times \overset{n}{\underset{j}{\sum }}ND_{ij}\;i\neq j$$

where $PD_{ij}$ and $ND_{ij}$ denote phylogenetic and niche distance between species i and its neighbor j, and n is the number of neighbors belonging to species i. Further, we constructed 1000 random networks corresponding to empirical networks according to Erdos-Renyi model and generated a null distribution of $null.MNPD_{i}$ and $null.MNND_{i}$ values. The standardized effect scores of $ses.MNPD_{i}$ and $ses.MNND_{i}$ were given by:

(3) $$ses.MNPD_{i}=\frac{obs.MNPD_{i}-mean\left(null.MNPD_{i}\right)}{sd\left(null.MNPD_{i}\right)}$$

and:

(4) $$ses.MNND_{i}=\frac{obs.MNND_{i}-mean\left(null.MNND_{i}\right)}{sd\left(null.MNND_{i}\right)}\;$$

To further explore the design principles of ecological networks at the motif level, we calculated the preferences in phylogeny and niche of motif position j (j=1,2,3…,148) by:

(5) $$ses.MNPD_{j}=\underset{i}{\sum }(f_{ij}\times ses.MNPD_{i})\;$$

and:

(6) $$ses.MNND_{j}=\underset{i}{\sum }\left(f_{ij}\times ses.MNND_{i}\right)$$

where $f_{ij}$ is the frequency of the node i in motif position j ($\sum_{i}f_{ij}=1$). As for the module level, the preferences in the phylogeny and niches of module k were given by:

(7) $$ses.MNPD_{k}=\frac{\sum_{i}\left(\lambda_{ij}\times ses.MNPD_{i}\right)}{\sum_{i}\lambda_{ij}}$$

and:

(8) $$ses.MNND_{k}=\frac{\sum_{i}\left(\lambda_{ij}\times ses.MNND_{i}\right)}{\sum_{i}\lambda_{ij}}$$

where $\lambda_{ij}$ is the probability of node i clustering into module k.

We tested the linear relationships between the phylogenetic or niches distance and network path distance with ‘lm’ functions. Further, a random forest algorithm [47] was used to classify the network path distance based on species phylogeny and niche distance. Finally, we calculated the Pearson’s correlations between phylogeny or niches distance and latent space distance across network path distance. These analyses were performed with packages of ‘base’ and ‘randomForest’ V4.6–14.

### 2.4. Mathematical Mechanism Model

#### 2.4.1. The dynamic of a Scale-Free Network in Ecology Community

Most of ecological networks were generally self-organized and exhibited scale-free property, such as food webs [48,49], species co-occurrence [20,50] and pollination networks [51,52]. In scale-free networks, the probability p of a vertex with certain k links (or degree) followed a power law distribution [33,34,53]:

(9) $$p\left(k\right)=k^{-\gamma }$$

where γ is an attraction factor. A greater γ means the weaker ability of a node to attract other nodes [54]. The value of γ < 3.47 holds for almost all of empirical networks, and the most of these are range from 2 to 3 [53,55].

**Figure 2:**
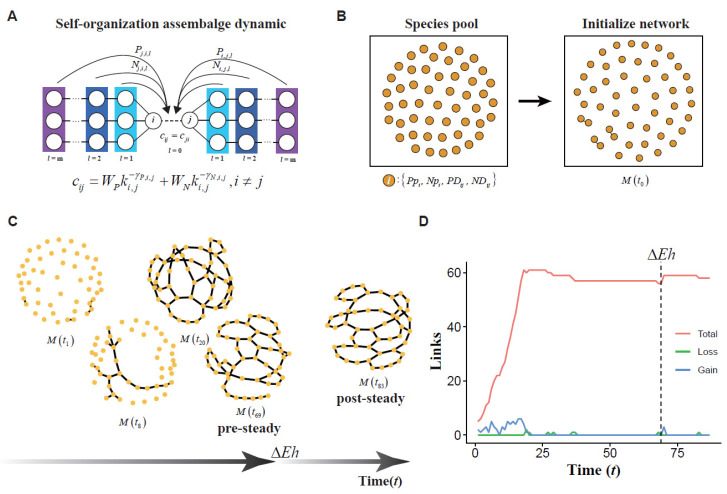
The simulation of self-organized assemblages in microbial networks. (**A**) The self-organized assemblages of microbial networks constrained by species phylogeny and niches. The connectivity *c_ij_* between species *i* and *j* is constrained by phylogeny (*P*) and niches (*N*). These two constraints could be directly affected by this pair of species (*l* = 0) and their neighbor (*l* = 1) or non-neighbor species (*l* > 1). (**B**) Characteristics of species pool and network initialization. In species pool, species *i* has preferences in phylogeny (*Pp_i_*) or niche similarity (*Np_i_*). The phylogeny and niche distance between species *i* and *j* are denoted by *PD_ij_* and *ND_ij_*, respectively. For network initialization, three pairs of species are randomly selected and linked. The initialized network is denoted by symmetrical adjacent matrix *M* (*t* = 0). (**C**) The simulation of self-organized assemblages in microbial networks. Symmetrical adjacent matrix *M* (*t*) was repeatedly updated according to network assembly procedures until termination events were triggered (See details in Supplementary Material) and the complete assembly of a pre-steady network. When environment heterogeneity changes (Δ*Eh*), *M* (*t*) becomes involved in iterations until iteration termination and achieves a post-steady state. (**D**) The link dynamic in network assembly process. The red line is the number of network links across assembly time. The green and blue lines are the number of loss and gain links in assembly processes.

To explore the assemblage dynamic of microbial co-occurrence networks, we took evolutionary and ecological processes into account and extended the above formula (Figure 2A). The connectivity strength $c_{ij}$ between species i and j is given by:

(10) $$c_{ij}=W_{P}k_{i,j}^{-\gamma_{P,i,j}}+W_{N}k_{i,j}^{-\gamma_{N,i,j}}i\neq j;\;i,j\;\in V$$

where $W_{P}$ and $W_{N}$ are the weight of preferences for the similarity of phylogeny and niches to $c_{ij}$, respectively, and $k_{i,j}$ is the average degree of i and j. The parameters of $\gamma_{P,i,j}$ and $\gamma_{N,i,j}$ are the mutual attraction factors between i and j driven by phylogeny and niche similarity, as described by following formula:

(11) $$\gamma_{P,ij}=-a+\overset{}{\underset{l}{\sum }}w_{P,l}\times P_{l}$$

and:

(12) $$\gamma_{N,ij}=-a+\overset{}{\underset{l}{\sum }}w_{N,l}\times N_{l}$$

where a is a constant representing the original attraction between species, $w_{P,l}$ and $w_{N,l}$ are the weights of the preference of phylogeny and niches similarity among species across network path distance l. If l=0, $w_{P,0}$ and $w_{N,0}$ reflect the direct phylogeny ($P_{0}$) and niche ($N_{0}$) attraction, respectively, originating from the preferences for phylogeny and niche similarity between i and j. If l>0, $w_{P,l}$ and $w_{N,l}$ reflect the weight of the indirect phylogeny ($P_{l}$) and niche ($N_{l}$) attraction, respectively; attraction received from neighboring (l=1); and the non-neighboring (l>1) vertexes with a distance of l to i or j.

We assumed that $w_{P,l}$ and $w_{N,l}$ accorded with an exponential attenuation model:

(13) $$w_{P,l}=\frac{A_{P}}{1+\exp\left(B_{P}\times \left(l+1\right)\right)}\;\;\;l=0,\;1,\;2,\ldots ,m$$

and:

(14) $$w_{N,l}=\frac{A_{N}\times \left(1+Eh\right)}{1+\exp\left(B_{N}\times \left(l+1\right)\right)}\;\;\;l=0,\;1,\;2,\ldots ,m\;$$

where $A_{P}$ and $A_{N}$ reflects the relative importance of phylogeny and niches, respectively. $B_{P}$ and $B_{N}$ is the attenuation rate of weight across network path distance l, Eh (Eh>0) is the degree of environment heterogeneity. Then, $W_{P}$ is given by:

(15) $$W_{P}=\underset{l}{\sum }w_{P,l}/\underset{l}{\sum }(w_{P,l}+w_{N,l})$$

and:

(16) $$W_{N}=1-W_{P}\;$$

The mutual attractions between pair nodes driven by phylogeny ($P_{l}$) and niches ($N_{l}$) in network path distance l are given by following formula:

(17) $$P_{l}=\frac{1}{2}\left(P_{i,j,l}+P_{j,i,l}\right),\;\;P_{i,j,0}=P_{j,i,0}$$

and:

(18) $$N_{l}=\frac{1}{2}\left(N_{i,j,l}+N_{j,i,l}\right),\;\;N_{i,j,0}=N_{j,i,0}$$

where $P_{i,j,0}$ and $N_{i,j,0}$ represent the direct mutual attractions of phylogeny and niches between i and j, respectively, given by:

(19) $$P_{i,j,0}=\frac{1}{2}\times \frac{Pp_{i}+Pp_{j}}{PD_{ij}}$$

and:

(20) $$N_{i,j,0}=\frac{1}{2}\times \frac{Np_{i}+Np_{j}}{ND_{ij}}$$

where $Pp_{i}$ and $Np_{i}$ denote the preference of species i in phylogeny and niche similarity, respectively. The phylogenetic and niche distances between i and j are represented by $PD_{ij}$ and $ND_{ij}$, respectively. Considering the indirect effects on the co-occurrence of i and j, the phylogenetic and niche attractions for i or j from non-neighbor species of j or i with a distance of l are given by Formulas (19) and (20), respectively, as follows:

(21) $$P_{i,j,l}=\frac{1}{2}\times \left(\frac{1}{q}\times \overset{q}{\underset{k=1}{\sum }}\frac{Pp_{\left(k,i,l\right)}+Pp_{j}}{PD_{\left(k,i,l\right)j}}+\frac{1}{p}\times \overset{p}{\underset{o=1}{\sum }}\frac{Pp_{\left(o,j,l\right)}+Pp_{i}}{PD_{\left(o,j,l\right)j}}\right)$$

and:

(22) $$N_{i,j,l}=\frac{1}{2}\times \left(\frac{1}{q}\times \overset{q}{\underset{k=1}{\sum }}\frac{Np_{\left(k,i,l\right)}+Np_{j}}{ND_{\left(k,i,l\right)j}}+\frac{1}{p}\times \overset{p}{\underset{o=1}{\sum }}\frac{Np_{\left(o,j,l\right)}+Np_{i}}{ND_{\left(o,j,l\right)j}}\right)$$

where k≠j, o≠i, k,o ϵV. (k,i,l) and (o,j,l) is the aggregations of neighbor or not-neighbor nodes belonging to i and j with distance l, respectively. The size of (k,i,l) and (o,j,l) are q and p, respectively.

#### 2.4.2. The Specie Pool Construction

In order to obtain the aggregation of species for ecological network simulations, we generated species pools with regard to phylogeny and niches (Figure 2B). The community phylogeny was constructed by a random phylogenetic tree according to the Paradis’s algorithms [56], and the branch length was generated from uniform distribution U(0,1). The niche distance between species i and j is given by:

(23) $$ND_{ij}=cPD_{i,j}+\varepsilon \;\mathrm{for}\;PD_{ij}<PS$$

and:

(24) $$ND_{ij}=U\left(\beta_{1}cPS,\beta_{2}cPS\right)+\varepsilon \;\mathrm{for}\;PD_{ij}\;\geq PS;\;\beta_{1}<\beta_{2}$$

where PS is phylogeny signal in the optimal niche, which is a threshold of the phylogenetic distance for the linear relationship between phylogenetic and niche distance. The parameters of c, $\beta_{1}$ and $\beta_{2}$, are constant, and ε is noise sampled from the normal distribution N(0, 0.01). Finally, pairwise phylogenetic distance $PD_{ij}$ and niche distance $ND_{ij}$ are standardized by Z-score transformation.

#### 2.4.3. Modeling of Network Assembly

At time $t_{0}$, the species pool with v species and a symmetrical adjacency matrix $M_{v\times v}\left(t=0\right)$ ($m_{i,j}=m_{j,i},\;m_{i,j}\in 0,\;1$) with e(t=0) links is generated. Additionally, we then repeated the following five steps until there were no loss and gain links among nodes or the number of iterations reached the maximum value. The brief procedures are shown in Figure 2.

Step 1: At time t, existing species i in networks are likely to interact with new introduced species or other existing species that have no interaction with species i. Regarding this, through Formula (25), we first calculated the pre-connectivity strength $pc_{i,j}$ between connected node i and its non-neighbor node j with a pre-average degree of i and j: $pk_{i,j}=k_{i,j}\left(t\right)+1$:

(25) $$pc_{i,j}=W_{p}pk_{i,j}^{-\gamma_{p,i,j}}+W_{h}pk_{i,j}^{-\gamma_{h,i,j}}$$

Step 2: To restrict the number of novel interactions generated at time t, we define a threshold $pc_{th}$ that is a quantile of $pc_{ij}$, with a given probability $p_{cth}$ and a minimal threshold $pc_{min}^{con}$. If $pc_{ij}$ > $\min\left(pc_{th},\;pc_{min}^{con}\right)$, node i would prepare to connect node j with probability $\min\left(pc_{ij},1\right)$ through a binomial experiment. After that, a pre-connectivity adjacency matrix $M_{con}$ is created.

Step 3: Then, we generate a transitive adjacency matrix $M_{tra}$ through:

(26) $$M_{tra}=M\left(t\right)+M_{con}$$

Step 4: After the introduction of novel species or interactions, original network patterns are changed and some existing interactions between species are weakened and may disconnect. Thus, we calculated the connectivity strength matrix $C_{tra}$ based on $M_{tra}$ through Formula (25), and then defined a threshold of connectivity strength $pc_{min}^{dis}$ for disconnection. If $c_{tra,i,j}$ < $pc_{min}^{dis}$, the link between i and j would disconnect with a probability $p_{dis}$ through the binomial experiment. After that, a pre-disconnected matrix $M_{dis}$ was created.

Step5: Finally, we obtained the new adjacency matrix at time t+1 through:

(27) $$M\left(t+1\right)=M_{tra}-M_{dis}$$

#### 2.4.4. Sensitive Analysis

We conducted a sensitivity analysis for the network assemblage model by Gaussian generalized linear model (GGLM) and random forest classification. First, the importance of phylogeny was set as twice as niche in the network assemblage, and the other of parameters varied within the given ranges (Table S1). Then, we varied the rest of parameters and generated 100 sets. For each parameter set, we sampled 100 replicates from modeling simulations. Finally, we defined the sensitivity of modeling outcomes to parameters as the regression coefficients of GGLM, where the dependent variables were the contributions of phylogeny and niche distance to network path distance given by random forest classification, and the independent variables were variable parameters.

To simulate the response of networks in the changes of environment heterogeneity, we examined the effects of the indirect interactions on the response of network dynamics in environment heterogeneity. In this model, environment heterogeneity is represented by Eh, which alters the weight of the contributions of phylogeny and niches to the probability of species co-occurrence. After completion of pre-steady network G(V,E) with presetting Eh (default Eh=0), Eh was increased to 2 and adjacent matrix of G was repeatedly updated. When the iterations achieved termination, we obtained a post-steady network $G^{\prime }\left(V^{\prime },E^{\prime }\right)$. Finally, we calculated link turnover, including the percentages of link gain (G) and loss (L) by following formula:

(28) $$G=\frac{\left|E_{g}\right|}{\left|E\right|}\times 100\%,\;E_{g}\subseteq E^{\prime },\;E_{g}\cap^{\;}E=Ø$$

and:

(29) $$L=\frac{\left|E_{g}\right|}{\left|E\right|}\times 100\%,\;E_{g}\subseteq E,\;E_{g}\cap^{\;}E^{\prime }=Ø$$

where |E| represents the number of links in pre-steady network.

## 3. Results

**Figure 3:**
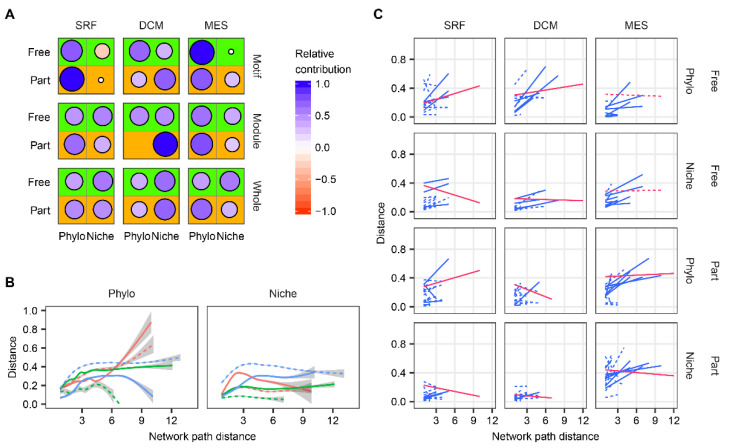
The effects of species phylogeny and niches on marine microbial network constructions. (**A**) The relative contributions of species phylogeny (Phylo) and niche distance to network construction across motif, module and whole-network (whole) levels in free-living (Free) and particle-associated (Part) communities at surface water layer (SRF), deep chlorophyll maximum layer (DCM) and mesopelagic zone (MES), where pattern size and color intensity are the strength of relative contribution, and colors represent the positively (blue) or negatively (red) relative contributions. (**B**) The relationships between network path distance and phylogeny or niche distance at the whole network level, where color represent depth layers (red: SRF; green: DCM blue: MES), and line type represents community fractions (solid: Free; dashed: Part). The curves were fitted by generalized additive models. The shaded region indicates standard errors of the curves. (**C**) The linear relationships between network path distance and phylogeny or niche distance across modules (blue line) or within the same modules (red line), where line type is the statistical significance of linear regression analysis (solid: *p* < 0.05).

Species phylogeny and niche similarity significantly correlated (*p* < 0.05) with, but showed differentiated contributions to, the marine microbial co-occurrence network structure at the motif, module and whole-network level (Figure 3A). Particularly, phylogeny made more contributions to network construction than niches at the motif level (Figure 3A; paired t-test, *p* = 0.07), while it was inconsistent at the module and whole-network level (Figure 3A; both *p* > 0.1). Specifically, at mesopelagic zone (MES), phylogeny, rather than niches, was a primary driver of particle-associated communities across three network levels (Figure 3A; *p* = 0.016), but not for free-living communities (Figure 3A). When the network path distance between species was considered, both phylogeny and niche distance positively correlated with network path distance up to around three, and then showed incongruent trends (Figure 3B). Such a phenomenon was further supported by the fact that phylogeny and niche distances were significantly (*p* < 0.05) positively related to network path distance within the same modules (Figure 3C), and these relationships were stronger than those across modules (Figure 3C). These results implied phylogeny and niche processes had strong constraints on network assemblage within a short range of network path distance, which may be particularly important for network module clustering.

**Figure 4:**
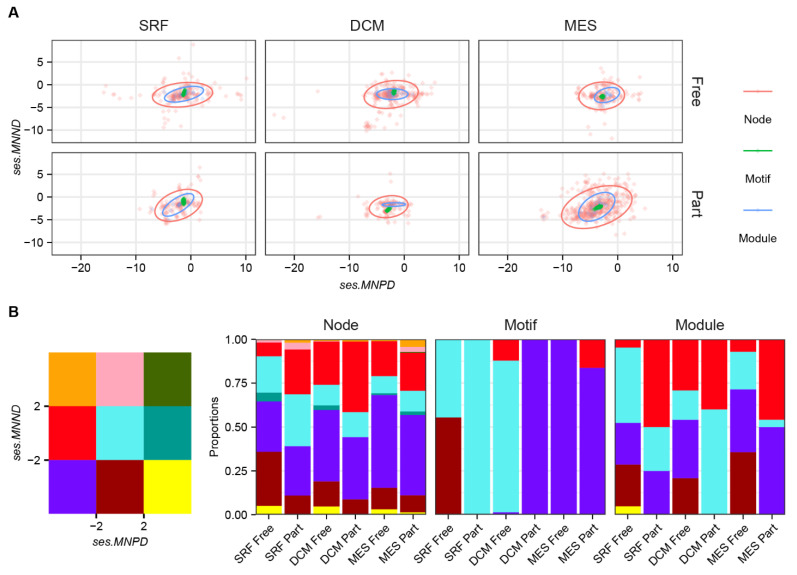
The mean neighbor phylogeny and niche distance of species in microbial networks. (**A**) The *ses.MNPD*–*ses.MNND* plot for marine microbial communities across node, motif and module levels, where *ses.MNPD* or *ses.MNND* of motifs and modules were calculated according to the proportions of their node members. (**B**) The block proportions in *ses.MNPD*–*ses.MNND* plots across nodes, motifs and modules, where a *ses.MNPD* (or *ses.MNND*) larger than 2 indicates that one object has a significant preference in a more distantly related phylogeny (or niche) at same network level, while a *ses.MNPD* (or *ses.MNND*) less than −2 indicates one object has significant preference in a more closely related phylogeny (or niche).

To determine whether species prefer to interact with phylogenetically close species or ecologically similar species, we developed novel indicators of mean neighbor phylogeny distance (*MNPD*)- and mean neighbor niche distance (*MNND*)-based ecological networks. Compared with the module level, nodes and motif positions showed the highest and lowest divergence, respectively, in *ses.MNPD*-*ses.MNND* plots (Figure 4A). Across six groups of microbial communities, over 95% of nodes showed a preference in phylogeny or niche-similar neighbors (*ses.MNPD* < 0 or *ses.MNND* < 0) and more than 65% of them showed a statistical significance (*ses.MNPD* < −2 or *ses.MNND* < −2; Figure 4B). For motif positions, all *ses.MNPD* and *ses.MNND* values were less than 0. This was especially true for the mesopelagic zone as all motif positions showed significant preferences in phylogeny or niche similarity: *ses.MNPD* or *ses.MNND* values < −2 (Figure 4B). These results indicate that phylogeny and niche processes have stronger constraints on network motifs or modules than on nodes. In addition, *ses.MNPD* had more significantly (*p* < 0.05) positive influences on node connectivity in particle-associated communities than in free-living ones, which were supported by the generalized linear model (Table 1).

**Figure 5:**
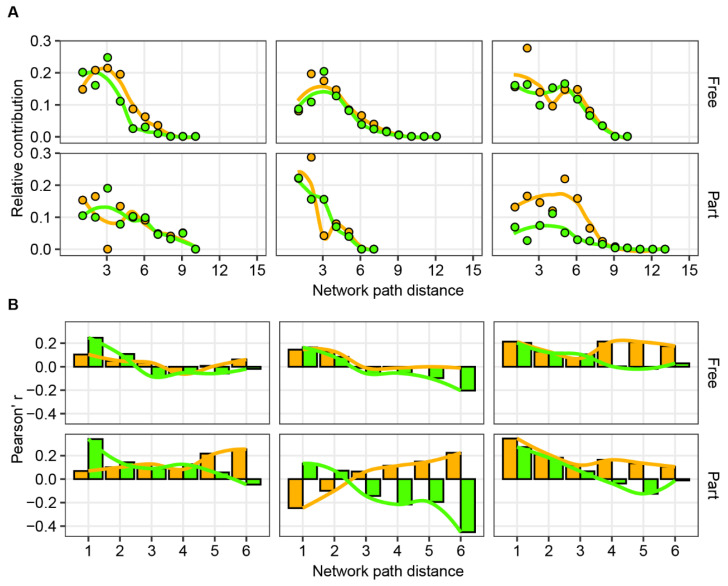
The contribution of species phylogeny and niche distance to their direct and indirect interactions. (**A**) The relative contribution of species phylogeny and niche distance to classifications of network path distance estimated by random forest classification analysis. The colors of points or lines vary with phylogeny (orange) and niche (green). The line is a Loess fit based on the points. (**B**) The Pearson’s correlations between species phylogeny or niche distance and network latent space distance ignore the potential for modularity across the network path distance. The colors of bars or lines vary with phylogeny (orange) and niche (green). The lines are a Loess fit based on the values of bars.

Moreover, a random forest classification analysis [47] showed that phylogeny and niche distance affected both direct and indirect species interactions, that is, network path distances = 1 and >1, respectively (Figure 5). Regarding a large network path distance, the contributions of phylogeny and niches for the network path distance between two species generally increased and then decreased with a network path distance of around six. Such patterns showed a peak in the network path distances from two to five (Figure 5A). Notably, the contribution of phylogeny was around two times higher than that of niches in particle-associated communities at MES, and showed stronger long-lasting effects on indirect species interactions (Figure 5A). The highest importance of phylogeny and niches to network path distance classification were 0.286 and 0.247, respectively (Figure 5A).

To further assess the influence of phylogeny and niches on indirect species interactions, we calculated the latent space distance between species with a variational Bayes method [41] and estimated the Pearson’s coefficients between the distance matrices of latent space and phylogeny or niches across the network path distance. We found that species phylogeny or niche distance significantly correlated with the latent space distance (*p* < 0.05; Figure 5B), and these correlations generally decreased with increasing network path distance when ignoring modularity (Figure 5B). Interestingly, when including modularity, the constraints of phylogeny on species latent distance in ecological networks weakened. This is especially true for the particle-associated communities in the deep chlorophyll maximum layer (DCM; Figure S1B) and there were no significant (*p* > 0.05) correlations between species phylogenetic and latent space distances.

To disentangle the ecological significance of the observed indirect species interactions from the perspective of network stability, we established a mathematical model based on scale-free network and simulated the processes of microbial co-occurrence network self-organized assemblages (Figure 2). In a sensitive analysis of model parameters, our results showed that the distance of long-lasting effects *l* had a greater influence than the other parameters, such as community size *S* and attraction constant *a*, regarding the contribution of phylogeny and niche to indirect interactions, particularly in long-distance indirect interactions (Tables S2 and S3). In addition, the strength of the phylogeny signal in the niche optima of species significantly (*p* < 0.05) enhanced the contribution of niches to direct interactions in the community assemblages, primarily driven by phylogeny (Table S3).

**Figure 6:**
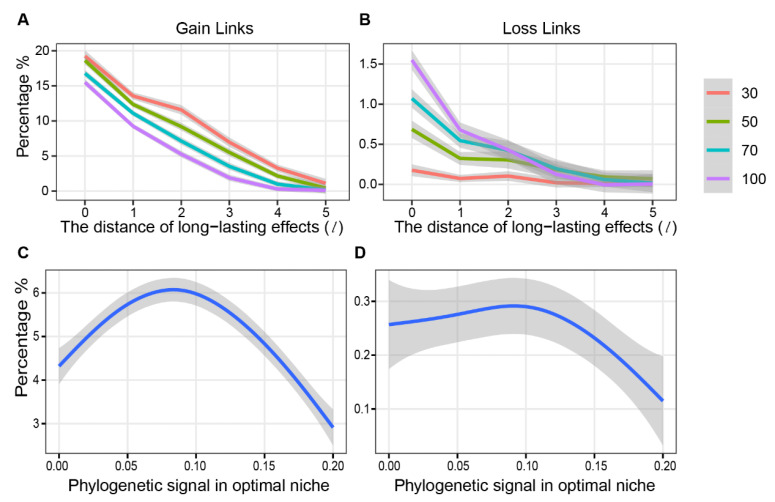
The response of network links in the changes in environmental heterogeneity. The gain links (**A**) and loss links (**B**) of the network across the distance of long-lasting effects (*l*) and community size (*N*; labeled with different colors) when environmental heterogeneity changes. The lines are a Loess fit based on 600 iteration points (6 × 100 replicates). The shaded regions indicate standard errors of the Loess curves. The gain links (**C**) and loss links (**D**) of the network across the phylogenetic signal in optimal niches (here, *l* = 2 and *S* = 50). The lines are fitted by generalized additive models (GAM) based on 10,000 iteration points (100 × 100 replicates). The shaded regions indicate standard errors of the curves of GAM.

We further simulated the response of the ecological network in environment heterogeneity (defined as continuous variable Eh), which could decease the weight of phylogeny preference on the probability of species co-occurrence [57]. After completeness of pre-steady network assemblage, Eh was shifted from 0 to 20 and the post-steady network and was completed until the iterations of the matrix of connection strength terminated again (Figure 2C). To estimate network stability, we then calculated the link turnover [58], that is, the percentage of the gain and loss links. When species indirect interactions were considered in network construction, the ecological network had a better stability in more heterogeneous environments. Specifically, towards the long distance of long-lasting effects *l*, the percentage of gain (Figure 6A) and loss (Figure 6B) links reduced. Further, the influences of long-lasting effects on network stability in reducing link turnover were stronger in large, as opposed to small, communities (ANOVA *p* < 0.05). Moreover, the percentage of gain links showed unimodal patterns along the gradient of phylogenetic signals in optimal niches, with a peak at around 0.08 (Figure 6C). For the percentage of loss links, however, there was a minor variation until the phylogenetic signal increased up to around 0.01, and then it decreased rapidly (Figure 6D).

## 4. Discussion

The evolutionary and ecological processes on direct [5,6,8,59,60] and indirect species interactions [1,61,62,63,64] attracted extensive attention; however, the relative importance of these two processes on ecological networks and its assemblage dynamics are less understood. Here, we clearly showed that species interactions were evolutionarily and ecologically constrained in marine microbial co-occurrence networks across the four levels of nodes, motifs, modules and whole networks. We further found that evolutionary processes revealed stronger long-lasting effects on indirect species interactions than ecological processes and dominated the network assemblage in spatially homogeneous but microscopically heterogeneous environments, such as particle-associated communities in MES. Finally, we simulated the assemblage dynamics of microbial co-occurrence networks with mathematically modeling and revealed that, regarding large network path distances, long-lasting effects improved network stability, as indicated by the decreasing link turnover.

We found that evolutionary and ecological processes generally constrained direct and indirect species interactions in marine microbial co-occurrence networks. For direct interactions, the findings were consistent with previous reports on predator–prey networks and competition networks [11,60]. For instance, over 80% of the observed realized and unrealized predator–prey interactions, involving 20 ground beetles and 115 prey species, could be predicted with phylogeny and functional trait information [11]. We further found that node connectivity in ecological networks was also associated with phylogeny and niches, implying that there was a phylogenetical conservatism [65] or niche specialization [66] in species interactions.

For indirect interactions, the findings on the strong effects of the two processes were consistent with previous reports of empirical networks among trophic levels, such as plant–animal-mutualistic [67] or predator–prey networks [1]. For example, with regard to the evolutionary processes in mutualistic networks, it has stronger constraints on consumer levels than resource levels and maintains network stability via positive within-guild indirect interactions between phylogenetically related species [67,68]. As for ecological processes, species density and function traits mediate indirect species coexistence and coextinction in plant–insect communities [1,62,69]. However, these reports show that indirect species interactions merely happen among three or four trophic levels, while we highlight that the two species indirectly connected within an ecological network could be generally constrained by phylogeny and niches, no matter whether they belong to different trophic levels or not.

Species interactions are fundamental for the assembly of ecological networks, and we expect that the assembly of the co-occurrence network is also driven by evolutionary and ecological processes. However, the ways that co-occurrence network assembly is driven by these two processes is a top-level issue, and could help us to essentially understand the self-organization of the microbial co-occurrence network. With integrations of empirical results, we proposed three reasonable principles of co-occurrence network assembly in marine microbial communities.

First, the phylogeny and niche are the primary drivers used to build the basic network blocks, such as motifs. For species, our null model analyses suggested that over 74% of species preferred to co-occur with phylogeny- or niche-related species, which was strongly supported by previous studies [20,26,28,70]. For motifs, all motif positions displayed the accordant preferences in phylogeny and niche similarity, which revealed that evolutionary and ecological processes had strong influences on building network motifs. It should be noted that, here we did not further consider that the effects of phylogeny and niches may vary regarding the type and strength of species interactions [10,12,71,72,73,74].

Second, both drivers had long-lasting effects on indirect species interactions within co-occurrence networks, and the strength of such effects decayed toward large network path distances. The long-lasting effects observed here are that the phylogeny and niche distance of pair species partly contribute to their indirect interactions, despite the large network path distance between them. Such findings agree with previous studies on food webs [12,63,75]. Furthermore, we revealed that the strength of long-lasting effects generally weakened with the increasing network path distance. Interestingly, the long-lasting effects of phylogeny and niches mostly occurred within a network path distance of around six, which may explain the phenomenon that the long-lasting effects were stronger within the same modules than across different modules. Overall, these long-lasting effects are short-range and could be the key mechanisms to enhance network modularity.

Third, phylogeny was more important than niches in determining direct or indirect species interactions when spatial homogeneity was greater. As marine depth increased and spatial homogeneity increased, the relative contributions of phylogeny to network constructions increased, while niches decreased (Figure 3A). For example, in the mesopelagic zone and surface water layer, phylogeny dominated the network assembly of particle-associated communities. However, free-living communities in the mesopelagic zone have no such phenomenon, which reveals that the microscale stability provided by particle may further strengthen the effects of phylogeny on the co-occurrence network assembly.

Based on these three principles, we proposed a mathematical model based on a scale-free network and simulated the self-organization of the co-occurrence network, which was key to exploring the network properties preceding empirical evidence. For example, regarding large network path distance, the long-lasting effects of both processes reduced link turnover and gave the network a strong resistance to environmental disturbances, indicating that the effects of indirect interactions on network stability may be more important than direct interactions [76]. This is particularly true for trophic networks with a low connectivity and high modularity [77] and for mutualistic networks with a nested architecture [78,79]. Moreover, the strong phylogenetic signals in optimal niches stabilized the successions of the co-occurrence network as expected when the determinants of network assembly shifted from evolutionary to ecological processes [80] (Figure 5B). However, for weaker phylogenetic signals, the percentage of gain links increased with the increasing phylogenetically signal. This might be caused by the increasing level of species connectivity with a higher strength than the minimum threshold of connections. This priori knowledge highlights that network stability should be further explored from design principles, except for network topologies such as size [81], connections [82] and modularity [83], because the latter are post analytical results and the outcomes of the former. Finally, the contributions of phylogeny and niches to direct and indirect species interactions had a great variety despite being from the same set of parameters, and this variety was greater for large network path distances (Figure S2). On the one hand, this supported multi-stability as the mechanism gave rise to different communities under the same environmental conditions [84]. On the other hand, it also indicated that stochastic processes may greatly affect the contributions of phylogeny and niches to network assembly in remotely indirect interactions.

Here, although we show a reasonable picture on how species phylogeny and niches shape microbial co-occurrence networks, three major caveats could be acknowledged regarding the analytical framework. First, microbial co-occurrence networks were inferenced by the correlation detection technique, which would lead to reasonable false positive rates [39], although we did not consider extremely rare species in network constructions. Meanwhile, specific ecological relationships, such as predation, competition, symbiosis and mutualism, could not be further accessed, and these ecological networks may have different and novel findings. Second, there are many correlation detection methods used to construct microbial networks, such as CoNet [85], RMT [86], MIC [87], LSA [88] and FlashWeave [89], which consistently produce different numbers and types of significant species correlation for the same data. Here, we used the SparCC tool for co-occurrence network constructions because it performed well in compositional data and data sparsity [39] and had significant edges with high correlations for analyses, which could greatly avoid noise and better than other tools. Third, some direct species interactions could be driven by indirect species interactions through other intermediary species, which could not be completely detected by the current methods and thus would decrease the robustness of results.

## 5. Conclusions

We found that direct and indirect species interactions in marine microbial co-occurrence networks were evolutionarily and ecologically constrained across four network levels of node, motif, module and the whole network, resulting in the small-world phenomenon of ecological networks. Compared to ecological processes, evolutionary processes dominated network assemblages in spatially homogeneous environments and showed stronger long-lasting effects on indirect species interactions. The contributions of two processes decreased toward long network path distance, but such long-lasting effects played a key role in stabilizing network link turnovers under environmental disturbances. These results provide a novel insight into the self-organization and modularization of marine microbial co-occurrence networks and highlight the importance of species phylogeny and niches in ecological network assemblages.

## Figures and Tables

**Table 1:** The influences of *ses.MNPD* and *ses.MNND* on node connectivity were analyzed by the generalized linear model. SRF: surface water layer; DCM: deep chlorophyll maximum layer; MES: mesopelagic zone.

Size Fraction	Layer	*ses.MNPD*	*ses.MNND*
Free-living	SRF	0.133	−0.0236
	DCM	0.2302	0.8291 *
	MES	−0.1677	−0.2277
Particle-associated	SRF	0.7122 ***	−0.0927
	DCM	0.3903 **	−0.0811
	MES	0.1315	−0.7282 **

***: *p* < 0.001; **: *p* < 0.01; *: *p* < 0.05.

## Data Availability

The original sequences of 16S rRNA tags, OTU table and meta data are available on TARA Ocean website [90]. Codes and relevant data presented in this paper are available on a GitHub repository at https://github.com/zhenghualiu/_phylo_habitat_network (accessed on 19 March 2021).

## Supplementary Materials

The following supporting information can be downloaded at: https://www.mdpi.com/article/10.3390/biology11040592/s1, Figure S1: The Pearson’s correlations between species phylogeny or niche distance and latent space distance that include the potential for modularity; Figure S2: The coefficient of variation for the importance of phylogeny and niche to network connections toward large network path distance; Table S1: Default parameters for sensitive analysis; Table S2: Influences of model parameters on the explanation of phylogenetic distance for network path distance; Table S3 Influences of model parameters on the explanation of niche distance for network path distance.
