# Supplementary material for: The Self-Organization of Marine Microbial Networks under Evolutionary and Ecological Processes: Observations and Modeling
Source: Biology (Basel). 2022 Apr 13;11(4):592. doi: 10.3390/biology11040592 (PMC9031791; doi:10.3390/biology11040592)
Supplement: Supplementary file 1 [file biology-11-00592-s001.zip › biology-1675640-supplementary.pdf]

Supplementary tables and figures for

The Self-Organization of Marine Microbial Networks under Evolutionary and Ecological Processes: Observations and Modeling

The file includes:

Supplementary Tables S1−S3

Supplementary Figures S1−S2

Table S1. Default parameters for sensitive analysis.

Parameters	Scope
A_P, A_N	2,1
B_P, B_N	0.5,0.5
N	[50, 100]
L	[0, 4]
a	[2,3]
pc_{min}^{con}	[0.08,0.12]
pc_{min}^{dis}	[0.06,0.08]
pc_{th}	[0.95,0.99]
PS	[0.12,0.2]

A_P and A_N reflects the relative importance of phylogeny and niche, respectively. B_P and B_N is the attenuation rate of weight across network path distance l. N is the size of species pool and a is a constant representing original attraction between species. pc_{min}^{con} and pc_{min}^{dis} is the minimal strength of connection and disconnection, respectively. pc_{th} is the quantile threshold of connection strength and PS is the phylogeny signal in optima niche.

Table S2. Influences of model parameters on the explanation of phylogenetic distance for network path distance.

Network path distance	1	2	3	4	5	6	7	8
N	**−0.0009*** **	**−0.0005*** **	**−0.0006***	−0.0004	−0.0009	0.0001	0.0001	−0.0001
L	**−0.0059*** **	**−0.0052*** **	**−0.0140*** **	**−0.0223*** **	**−0.0094*** **	−0.0011	**0.0028***	**0.0036*** **
a	−0.0095.	**−0.0208*** **	**−0.0621*** **	**−0.0980*** **	**−0.0514*** **	**−0.0203***	−0.0005	0.0036
pc_{min}^{con}	0.1135	−0.0434	**−0.5176***	−0.4536.	−0.1582	0.1296	0.1818	0.1512.
pc_{min}^{dis}	−0.2543	0.0251	0.2665	−0.2953	−1.231**	−0.7715*	−0.2469	−0.0433
pc_{th}	**1.3814*** **	**0.4716*** **	**0.5457***	0.5038.	−0.0947	−0.0071	0.0818	−0.1315
PS	0.0749	0.0508	−0.0027	0.0257	0.0553	0.0336	0.0163	0.020

'***': $p < 0.001$; '**': $p < 0.01$; '*': $p < 0.05$.

Table S3. Influences of model parameters on the explanation of niche distance for network path distance.

Network path distance	1	2	3	4	5	6	7	8
N	**−0.0003*** **	**−0.0003*** **	**−0.0004** **	−0.0002	−0.0001	0.0002	0.00001	−0.0001
L	**−0.0022*** **	**−0.0011*** **	**−0.0088*** **	**−0.0111*** **	**−0.0053** **	−0.0001	**0.0032** **	**0.0043*** **
a	0.0005	**−0.0062*** **	**−0.0430*** **	**−0.0561*** **	**−0.0348*** **	−0.0129.	−0.0026	0.0071
pc_{min}^{con}	0.0032	−0.0435.	**−0.3396** **	**−0.3065***	−0.2508	0.0833	0.1548	0.1549
pc_{min}^{dis}	−0.0730	0.0522	0.0964	−0.4688	−0.7225*	0.0470.	−0.1495	−0.0936
pc_{th}	**0.4426*** **	−0.0267	0.1156	−0.1688	−0.3420.	0.1953	−0.0203	0.0412
PS	**0.1264*** **	−0.0234.	−0.0979	−0.0628	0.0101	0.0244	0.01785	−0.0015

'***': $p < 0.001$; '**': $p < 0.01$; '*': $p < 0.05$.

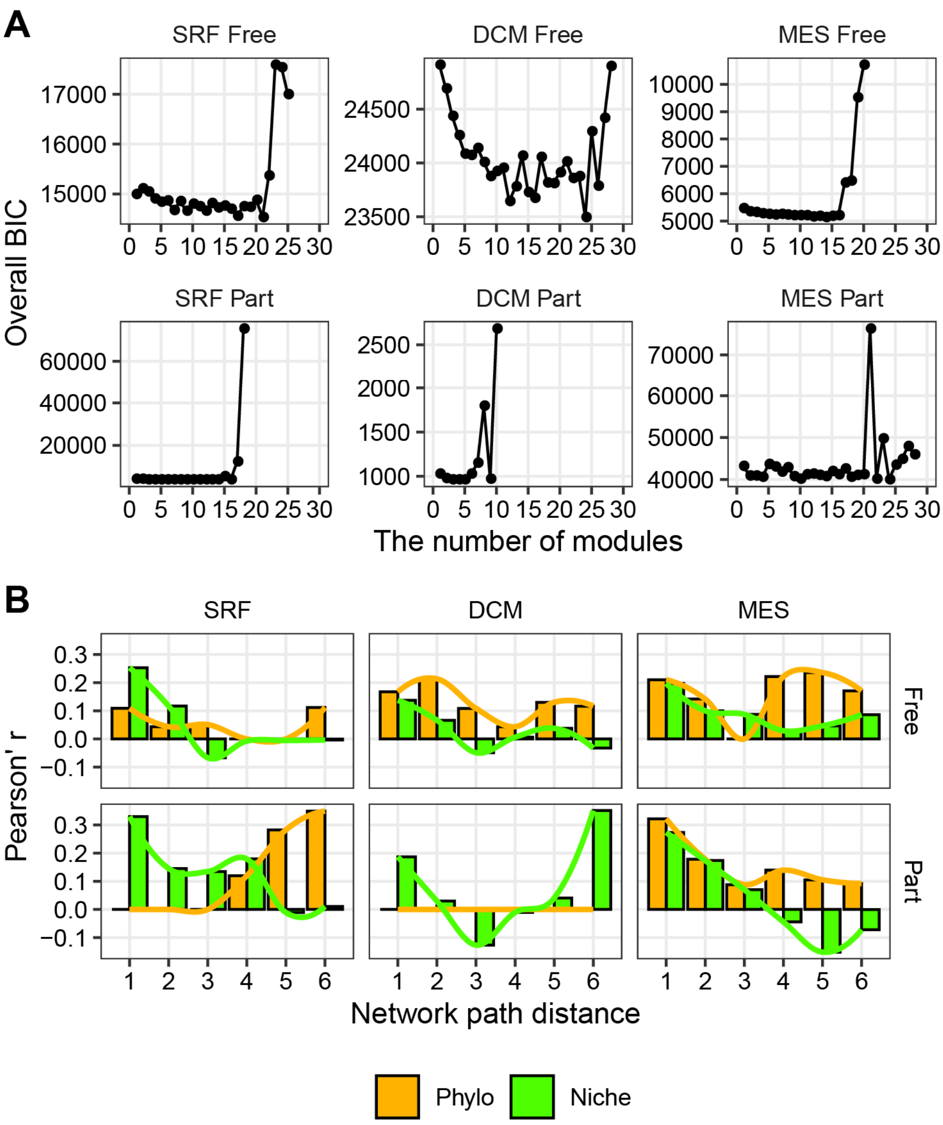

Figure S1. The Pearson's correlations between species phylogeny or niche distance and network latent space distance that include the potential for modularity. (A) The overall BIC of Variational Bayes Latent Position Cluster Model (VBLPCM) corresponding the setting parameter of potential module number, which was used to determine the best number of potential modules according to minimum overall BIC. **(B)** The Pearson's correlations between species phylogeny or niche distance and latent space distance including modularity across network path distance. The lines are loess fit based on the values of bars.

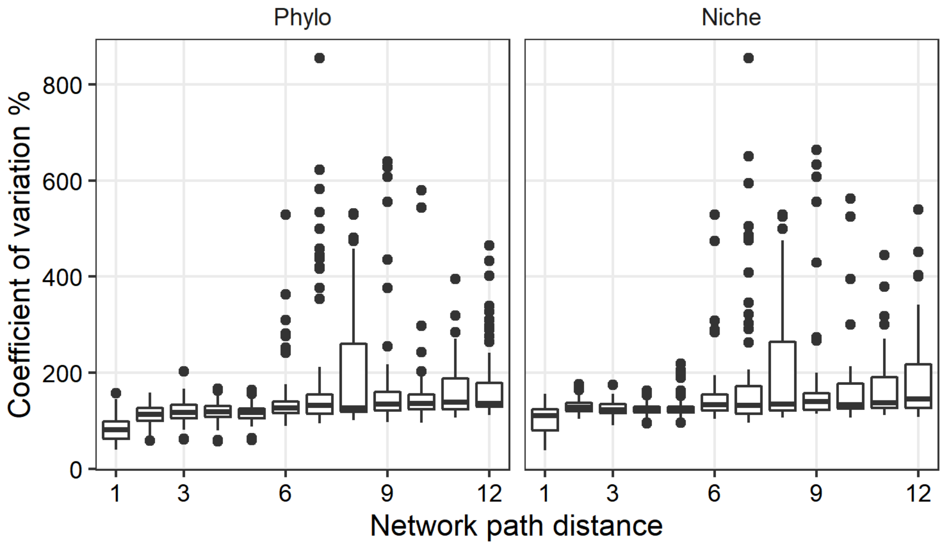

Figure S2. The coefficient of variation for the importance of phylogeny (Phylo) and niche (Niche) to network connections toward large network path distance.
